# Identification of Genes Required for Growth of *Escherichia coli* MG1655 at Moderately Low pH

**DOI:** 10.3389/fmicb.2016.01672

**Published:** 2016-10-25

**Authors:** Bram Vivijs, Abram Aertsen, Chris W. Michiels

**Affiliations:** Laboratory of Food Microbiology, Department of Microbial and Molecular Systems, and Leuven Food Science and Nutrition Research Centre (LFoRCe), KU LeuvenLeuven, Belgium

**Keywords:** *Escherichia coli*, acid stress, stress tolerance genes, genetic analysis, acidic foods

## Abstract

The survival of some pathotypes of *Escherichia coli* in very low pH environments like highly acidic foods and the stomach has been well documented and contributes to their success as foodborne pathogens. In contrast, the ability of *E. coli* to grow at moderately low pH has received less attention, although this property can be anticipated to be also very important for the safety of mildly acidic foods. Therefore, the objective of this study was to identify cellular functions required for growth of the non-pathogenic strain *E. coli* MG1655 at low pH. First, the role of the four *E. coli* amino acid decarboxylase systems, which are the major cellular mechanisms allowing extreme acid survival, was investigated using mutants defective in each of the systems. Only the lysine decarboxylase (CadA) was required for low pH growth. Secondly, a screening of 8544 random transposon insertion mutants resulted in the identification of six genes affecting growth in LB broth acidified to pH 4.50 with HCl. Two of the genes, encoding the transcriptional regulator LeuO and the elongation factor *P*-β-lysine ligase EpmA, can be linked to CadA production. Two other genes, encoding the diadenosine tetraphosphatase ApaH and the tRNA modification GTPase MnmE, have been previously implicated in the bacterial response to stresses other than low pH. A fifth gene encodes the LPS heptosyltransferase WaaC, and its mutant has a deep rough colony phenotype, which has been linked to reduced acid tolerance in earlier work. Finally, *tatC* encodes a secA-independent protein translocase that exports a few dozen proteins and thus is likely to have a pleiotropic phenotype. For *mnmE, apaH, epmA*, and *waaC*, de novo in frame deletion and genetic complementation confirmed their role in low pH growth, and these deletion mutants were also affected in growth in apple juice and tomato juice. However, the mutants were not affected in survival in gastric simulation medium at pH 2.5, indicating that growth at moderately low pH and survival of extremely low pH depend mostly on different cellular functions.

## Introduction

In the food production chain, bacteria encounter various environmental stresses, such as heat, cold, desiccation, oxidants, or acids. Acid stress, in particular, can have various sources, such as the natural acidity of fruits and fruit-based foods, the formation of organic acids in fermented foods and the addition of organic acids to acidify foods or as food preservatives. Moreover, after food ingestion, bacteria become exposed to the extremely acidic environment of the stomach, with a pH typically ranging from 1.0 to 2.5. The ability to adapt to environmental stresses allows bacteria to withstand the hostile conditions experienced in the food chain within certain limits. Adaptation to acid stress is especially relevant for infective foodborne pathogens. Indeed, in order to cause foodborne illness, these bacteria may not only become exposed to acid stress during food processing and storage, they also have to survive the harsh conditions in the stomach before they can infect the small intestine or the colon. Therefore, most studies related to acid stress in enteropathogens have mainly focused on the survival of important pathogens like *Salmonella* and enterohemorrhagic *Escherichia coli* (EHEC) upon extreme acid challenge for a few hours. These studies have revealed diverse mechanisms to survive extreme acid stress in these bacteria. One group of mechanisms is called acid resistance (AR), and is defined as the ability to survive an extreme acid challenge at pH 1.5–2.5 (Beales, 2004). AR mechanisms are active in *E. coli* but seem to be mostly lacking in *Salmonella*, which explains the lower level of AR in the latter. The other group of mechanisms is the acid tolerance response (ATR), and is defined as the ability to survive a normally lethal acid challenge at pH 3.0 – 3.5 after acid adaptation at moderately low pH. These mechanisms have been mainly studied in *Salmonella* (Álvarez-Ordóñez et al., 2012).

Besides passive cytoplasmic buffering, five active stationary-phase AR systems have been described in *E. coli* (Kanjee and Houry, 2013). The AR1 system is referred to as the oxidative or glucose-repressed system and is only poorly understood. This system is activated under oxidative growth conditions and allows *E. coli* cells to survive a subsequent acid challenge. This is usually achieved by growing cells to stationary phase in a glucose-free complex medium at pH 5.5, and by subsequently challenging them in a minimal medium, without an external supply of amino acid substrates, at pH 2.5. The other four AR systems (AR2-5) all rely on the activity of amino acid-dependent decarboxylase/antiporter systems and therefore require the presence of specific amino acids in the acid challenge medium. Each of these systems decarboxylates a specific amino acid (glutamate, arginine, lysine, or ornithine) to its corresponding amine (γ-amino butyric acid (GABA), agmatine, cadaverine, or putrescine, respectively) by a cytoplasmic pyridoxal-5′-phosphate (PLP)-dependent decarboxylase, and subsequently exchanges the decarboxylated product for new amino acid substrate via a cognate inner membrane-bound antiporter (Kanjee and Houry, 2013). The glutamate-dependent AR system (AR2) is by far the most potent AR system in *E. coli* and consists of the homologous inducible glutamate decarboxylases GadA and GadB, and the glutamate/GABA antiporter GadC. In addition to the amino acid decarboxylase systems, the formate hydrogen lyase (FHL) complex, the adenosine deaminase Add and the glutaminase YbaS were also shown to contribute to AR in *E. coli* by promoting proton consumption (Noguchi et al., 2010; Sun et al., 2012; Lu et al., 2013). The ATR in *Salmonella* Typhimurium induces the expression of a large number of genes and prepares cells for lethal acid challenge by the activation of homeostatic mechanisms that maintain or raise the intracellular pH, the production of acid shock proteins with a role in protecting or repairing damaged proteins and DNA, and modifying membrane composition to increase the barrier properties for protons (Álvarez-Ordóñez et al., 2012).

Compared to the survival of extremely low pH, less is known about the mechanisms supporting growth of *E. coli* and other *Enterobacteriaceae* at moderately low pH (pH 4.0–5.0), although these are at least equally important for food safety and food stability as the acid survival mechanisms. Several studies have reported growth of various members of the *Enterobacteriaceae* at surprisingly low pH values. For example, the pH_min_ of 188 *E. coli* strains in lysogeny broth (LB) acidified with hydrochloric acid ranged from 3.8 to 4.3 (Haberbeck et al., 2015). Acidification of media with organic acids rather than hydrochloric acid increases the pH_min_, but even in acidic foods containing organic acids, growth in this pH range has been reported. For example, strains of *E. coli* O157:H7 increased by 3.5 log cfu/ml in apple cider of pH 3.6 in 16 h at 23°C (Ukuku et al., 2009), and by 2.5 log cfu/ml in apple plugs of pH 3.3 in 24 h at 25°C (Alegre et al., 2010). In the context of food safety, it is important to note that the pH boundaries supporting growth are temperature dependent. From an extensive study of the interaction of temperature and pH on growth of *E. coli* it was concluded that at suboptimal pH values (down to pH 5.0) *T*_max_ decreased strongly, *T*_min_ decreased slightly and *T*_opt_ showed no clear trend (Baka et al., 2013). However, at pH values close to pH_min_, *T*_min_ was found to increase (Haberbeck et al., 2015). In an earlier work, it was found that pH_min_ of *E. coli* was constant between 25 and 37°C (Presser et al., 1998).

*E. coli* and other enterobacteria exhibit a transcriptional and translational response during growth at moderately low pH that induces mechanisms contributing to improved pH homeostasis, some of which overlap with the AR mechanisms. Several components of the electron transport chain which are coupled to proton translocation are upregulated during aerobic growth under mild acid stress (Maurer et al., 2005). The membrane potential that builds up as a result and that would impede sustained proton expulsion is dissipated by simultaneous uptake of K^+^. Further, the expression of enzymes that catalyze reactions which consume cytoplasmic protons, such as hydrogenases or amino acid decarboxylases, is increased under acidic conditions (Hayes et al., 2006). Interestingly, the products of lysine and ornithine decarboxylation (cadaverine and putrescine, respectively) also inhibit the OmpF and OmpC porins, thereby decreasing the outer membrane proton permeability (Dela Vega and Delcour, 1996). Growth at moderately low pH also enhances transport and metabolism of secondary carbon sources, such as sugars (e.g., ribose, arabinose, and fuculose) and sugar derivatives (e.g., galactitol, sorbitol, and gluconate) that produce fewer acids upon fermentation than glucose (Hayes et al., 2006). Finally, *E. coli* incorporates higher proportions of cyclopropane fatty acids during growth at low pH, which reduces the proton permeability of the inner membrane under mild acid stress and has also be linked with a slightly increased ability to extrude protons upon acid stress (Shabala and Ross, 2008).

The objective of the current work was to conduct a comprehensive genetic analysis to identify (novel) genes required for growth of *E. coli* at moderately low pH, and to determine whether these genes play a role in survival of extreme AR. First, the role of the known decarboxylase AR systems in growth at low pH was investigated, and subsequently a genome-wide screening for mutants deficient in low pH growth was conducted.

## Materials and Methods

### Bacterial Strains, Plasmids, and Oligonucleotides

Bacterial strains and plasmids used in this chapter are listed in **Table 1**. *E. coli* MG1655 *gadA/B* and *adiA* were obtained via P1 transduction from *E. coli* EF522 and *E. coli* EF1022, respectively (Castanie-Cornet et al., 1999). Gene deletions were *de novo* constructed via a protocol described by Baba et al. (2006) using primers listed in **Table 2**. In this way, in-frame deletions encompassing the central region of the open reading frame from the second codon through and including the eighth codon before the C-terminus were created, leaving the start codon and translational signal for possible downstream genes intact. All deletions were verified by PCR using inwardly oriented primers complementary to the region left and right of the deleted genes (**Table 2**), and sequence analysis of the PCR products. Complementation plasmids were constructed by ligation of open reading frames into a NcoI and XbaI cut pTrc99A plasmid under control of the inducible *P*_trc_ promoter, and correct insertion was verified using primers pTrc99A_seq_FW and pTrc99A_seq_REV.

**Table 1:** Strains and plasmids used in this chapter.

Strain or plasmid	Relevant features	Reference
***Escherichia coli* MG1655 strains**		
Wild-type	F^-^ λ^-^ *rph-1*	Guyer et al., 1981
*gadA/B*	*gadA*::pRR10 (Ap) *gadB*::Km, obtained via P1 transduction from *E. coli* EF522	This work
*adiA*	*adiA*::MudJ (Km), obtained via P1 transduction from *E. coli* EF1022	This work
*ΔcadA*	In-frame deletion of *cadA*	This work
*ΔspeF*	In-frame deletion of *speF*	This work
*ΔwaaC*	In-frame deletion of *waaC*	This work
*ΔleuO*	In-frame deletion of *leuO*	This work
*ΔmnmE*	In-frame deletion of *mnmE*	This work
*ΔapaH*	In-frame deletion of *apaH*	This work
*ΔepmA*	In-frame deletion of *epmA*	This work
**Plasmids**		
pTrc99A	Cloning vector carrying IPTG-inducible *trc* promoter (*P*_trc_); Ap^R^	Amann et al., 1988
pKD13	Template plasmid containing *kan* gene flanked by FRT sites; Km^R^ Ap^R^	Datsenko and Wanner, 2000
pKD46	Plasmid expressing γ, β, and *exo* recombination genes of phage λ under control of P_BAD_; temperature-sensitive replicon; Ap^R^	Datsenko and Wanner, 2000
pCP20	Plasmid expressing the FLP (flippase) gene, directing recombination of FRT sites; temperature-sensitive replicon; Ap^R^ Cm^R^	Datsenko and Wanner, 2000

**Table 2:** Primers used in this chapter.

Primer	Sequence (5′–3′)
**Primers for determination of transposon insertion site:**	
Linker 1	TTTCTGCTCGAATTCAAGCTTCTAACGATGTACGGGGACACATG
Linker 2	TGTCCCCGTACATCGTTAGAACTACTCGTACCATCCACAT
Y linker primer	CTGCTCGAATTCAAGCTTCT
NK_Cm_DWN	CCTCCCAGAGCCTGATAA
**Primers for deletions:**	
CadA_del_FW	TTTGTCCCATGTGTTGGGAGGGGCCTTTTTTACCTGGAGATATGACTATGATTCCGGGGATCCGTCGACC
CadA_del_REV	TGGCAAGCCACTTCCCTTGTACGAGCTAATTATTTTTTGCTTTCTTCTTTTGTAGGCTGGAGCTGCTTCG
SpeF_del_FW	TTCGAGAAATTGAGGACCTGCTATTACCTAAAATAAAGAGATGAAAAATGATTCCGGGGATCCGTCGACC
SpeF_del_REV	GACGCCCATTTTGTTCGATTTAGCCTGACTCATAATTTTTCCCCTTTCAATGTAGGCTGGAGCTGCTTCG
WaaC_del_FW	TACTGGAAGAACTCAACGCGCTATTGTTACAAGAGGAAGCCTGACGGATGATTCCGGGGATCCGTCGACC
WaaC_del_REV	AGTTTAAAGGATGTTAGCATGTTTTACCTTTATAATGATGATAACTTTTCTGTAGGCTGGAGCTGCTTCG
LeuO_del_FW	GCATTCCAATAAGGGAAAGGGAGTTAAGTGTGACAGTGGAGTTAAGTATGATTCCGGGGATCCGTCGACC
LeuO_del_REV	CATTCATGTCTGACCTATTCTGCAATCAGTTAGCGTTTGCAAATTGAGACTGTAGGCTGGAGCTGCTTCG
MnmE_del_FW	GGGCGGATAAGCACCGCGCATCCGCCACACAAAGCAACAGGAACATCATGATTCCGGGGATCCGTCGACC
MnmE_del_REV	ACAGTCAGAATGCGGCTTCGTAAGCGCGGTTACTTACCAATACAGAAGCTTGTAGGCTGGAGCTGCTTCG
ApaH_del_FW	TTCCCGTATTCCGACTCGCCGTTCCCACACTCATTCATTAAAAGAATATGATTCCGGGGATCCGTCGACC
ApaH_del_REV	TTATCCGGCCTTCCTATATCAGGCTGTGTTTAAGACGCCGCCGCTTCGCCTGTAGGCTGGAGCTGCTTCG
EpmA_del_FW	CGTTGCGAGTAGACTTCGTGCCCTTGTCAAAAACTGGAGATTTAACTATGATTCCGGGGATCCGTCGACC
EpmA_del_REV	TTCGCTGTTAATTCAGTAATTTTTCAGAATTATGCCCGGTCAACGCTAAATGTAGGCTGGAGCTGCTTCG
**Control primers:**	
CadA_ctr_FW	TCAATGGATAACCACACCGC
CadA_ctr_REV	AGAGAATGAGTAAGGCACGC
SpeF_ctr_FW	CCCTTGTATTATCAGCCACC
SpeF_ctr_REV	CCATACCGCCTGATTTACG
WaaC_ctr_FW	GCCCTGTATGGTCCGAGTAG
WaaC_ctr_REV	AGTAGCACGAAATGGCGAAT
LeuO_ctr_FW	AGACCGATAAAGCGAACGAT
LeuO_ctr_REV	GGCTCCAGACAACATCTCCA
MnmE_ctr_FW	CTGGGGCTTCTCCATTATCA
MnmE_ctr_REV	TCTAATCGGTTTGGCTCTGG
ApaH_ctr_FW	CAGGTTCAAAGCGTCTACAT
ApaH_ctr_REV	GGATGACTGGGAATCGGTAT
EpmA_ctr_FW	ACATCCTGCTCACACAACCA
EpmA_ctr_REV	CGCCTTCTTCTTTGCGATAA
pTrc99A_seq_FW	TGCAGGTCGTAAATCACTGC
pTrc99A_seq_REV	CTGGCAGTTCCCTACTCTCG
**Primers for complementation:**	
WaaC_NcoI	TAATCCATGGGACGGGTTTTGATCGTTAAAAC
WaaC_XbaI	CCGCTCTAGACCTTTATAATGATGATAACTTTTCC
MnmE_NcoI	TAATCCATGGGAAGCGATAATGACACTATCGTAG
MnmE_XbaI	ATGATCTAGAGCGCGGTTACTTACCAATAC
ApaH_NcoI	AGTACCATGGCGACATACCTTATTGGCGAC
ApaH_XbaI	AATTTCTAGATTAAGACGCCGCCGCTTCG
EpmA_NcoI	AAAACCATGGGAAGCGAAACGGCATCCTGG
EpmA_XbaI	CGAGTCTAGATTATGCCCGGTCAACGCTAA

### Screening for Mutants of *E. coli* MG1655 Affected in Growth at Moderately Low pH

A random knockout library of *E. coli* MG1655 was constructed using λNK1324, which carries a mini-Tn*10* transposon (1.4 kb) with a Cm resistance gene and a mutant transposase with relaxed target specificity, according to the protocol described by Kleckner et al. (1991). To maximize randomness and genome coverage, the mutant collection was assembled from 89 independent transposon mutagenesis experiments from each of which 96 mutants were isolated (i.e., a total of 8544 mutants). The mutants were stored in microplates at -20°C. To screen for growth at moderately low pH, the mutant library was first inoculated into microplates containing 300 μl LB broth (10 g/l tryptone, 5 g/l yeast extract, 5 g/l NaCl). These plates were covered with a foil and incubated overnight at 37°C. Then, the stationary-phase cultures were thousandfold diluted in microplates containing 300 μl LB acidified to pH 4.50 with HCl, and the plates were covered with a foil and incubated at 30°C for 24 h, when the OD_600_ was measured. Transposon insertion sites were determined using the method of Kwon and Ricke (2000). Briefly, genomic DNA of the mutants was isolated, digested with NlaIII and ligated with a Y-shaped linker, composed of oligonucleotides linker 1 and linker 2. Next, a PCR amplification was carried out using a transposon-specific primer (NK_Cm_DWN) and a primer specific to the Y-shaped linker (Y linker primer). The PCR product was subsequently sequenced using the transposon-specific primer and the insertion site was determined based on the known genome sequence of *E. coli* MG1655.

### Determination of Growth Curves Via OD Measurements

*Escherichia coli* strains were grown overnight to stationary phase in 4 ml LB at 37°C and thousandfold diluted in fresh LB acidified with HCl to the required pH. Culture volumes of 300 μl were transferred to the wells of a microplate, which was sealed with a cover foil and incubated in a Multiskan Ascent plate reader (Thermo Labsystems, Helsinki, Finland) at 30 or 37°C, and the OD_630_ was automatically measured every 15 min. The growth curves were fitted by the model of Baranyi and Roberts (1994), using the Excel add-in package DMFit (Institute of Food Research, Norwich, UK). For the decarboxylase mutants, growth curves were also recorded in a similar way in M9 medium (Sambrook et al., 1989) supplemented with 0.2% glucose, 0.1% casamino acids, and 1 mg/l thiamine, and adjusted to pH 4.80 with HCl, and to which 5 mM L-glutamic acid, L-arginine, L-lysine, or L-ornithine were added when required. In this case, wells were covered with 50 μl paraffin oil to create anoxic conditions required for decarboxylase activity.

### Determination of Growth Curves Via Plate Counts

Stationary-phase cultures, grown overnight in 4 ml LB at 37°C, were 100,000-fold diluted in test tubes containing 10 ml LB, apple or tomato juice that had been first adjusted to specific pH values with HCl or NaOH. The suspensions in LB were transferred to microplates (multiple 300 μl samples) and sealed with a cover foil. The test tubes with acidic juices and the microplates were incubated without shaking at 20 and 30°C, respectively. At regular time points, 300 μl samples were taken, serially diluted in potassium phosphate buffer (10 mM; pH 7.00) and subsequently spotted (5 μl) on LB agar. After 24 h of incubation at 37°C, the colony-forming units were determined.

### Acid Challenge (Survival) Assay

Stationary-phase cultures grown overnight at 37°C in 4 ml LB were hundredfold diluted in test tubes containing 4 ml of simulated gastric fluid (Beumer et al., 1992). The composition of this medium was 8.3 g/l bacteriological peptone, 3.5 g/l glucose, 2.05 g/l NaCl, 0.6 g/l KH_2_PO_4_, 0.11 g/l CaCl_2_, 0.37 g/l KCl, 0.05 g/l bile salts (Oxoid, Basingstoke, UK), 0.1 g/l lysozyme (66200 U/mg, Fluka, Buchs, Switzerland), and 13.3 mg/l pepsin (47 U/g, Fluka, Buchs, Switzerland), and the pH was adjusted to 2.50 with HCl. The suspensions were incubated at 37°C for 6 h. Every hour, 100 μl samples were taken, serially diluted in potassium phosphate buffer (10 mM; pH 7.00) and subsequently plated on LB agar. D-values (time required for a 10-fold reduction in viable cells) were determined by identification of the log-linear Bigelow model (for the Δ*waaC* mutant) or the log-linear model with shoulder (for the wild-type and other deletion mutants) using GInaFiT (Geeraerd et al., 2005).

### Statistical Analysis

All experiments were carried out in triplicate using cultures grown from different colonies on a single agar plate. Mean values of different strains or treatments were compared by Student’s *t*-test and differences were considered significant when a *p*-value of <0.05 was obtained.

## Results

### Role of *E. coli* Amino Acid Decarboxylase Systems in Growth at Moderately Low pH

**FIGURE 1:**
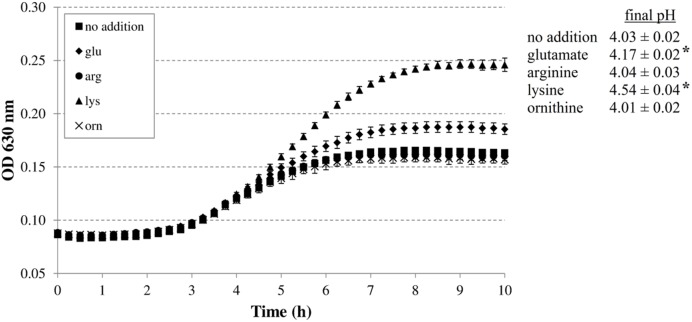
**Effect of supplementation with 5 mM glutamate, arginine, lysine, or ornithine on growth of *Escherichia coli* MG1655 at 37°C under anoxic conditions in M9 medium containing 0.2% glucose, 0.1% casamino acids, and 1 mg/l thiamine.** The initial pH of the medium was 4.80. Error bars represent standard deviations of three replicate cultures. The asterisks indicate when the final pH value was significantly different (*p* < 0.05) from the final pH value reached with no specific amino acid addition.

First, we investigated whether the addition of the four amino acid substrates of the AR systems 2, 3, 4, and 5 (glutamic acid, arginine, lysine, and ornithine, respectively) improves growth of *E. coli* MG1655 at a low initial pH. This was done in M9 minimal medium to reduce the basal levels of these amino acids compared to rich media like LB. However, 0.1% casamino acids were added to stimulate growth. The initial pH of the medium was set at 4.80, and final pH values were recorded as an indication of decarboxylase activity (**Figure 1**). The experiments with the decarboxylase mutants were conducted at 37°C. Although this temperature is not very relevant to most conditions at which foods are stored, it is more challenging for growth of *E. coli* at low pH than 30°C, and therefore expected to allow a more sensitive detection of any deficiencies in the mutants’ capacity to grow at low pH (see below).

**FIGURE 2:**
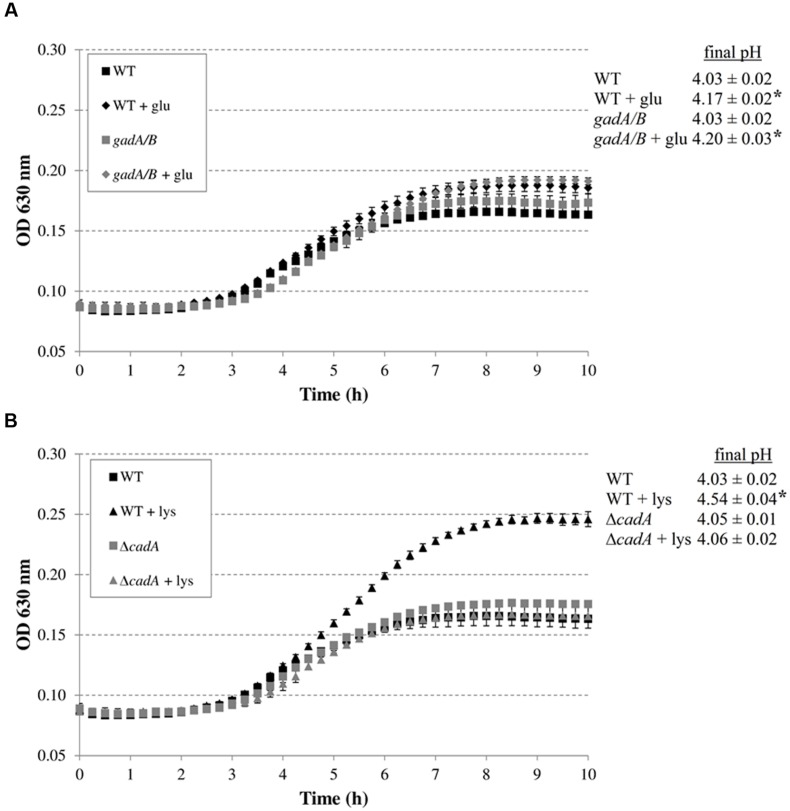
**Effect of supplementation with amino acids (5 mM) on growth of decarboxylase-deficient mutants of *E. coli* under anoxic conditions at 37°C in M9 medium containing 0.2% glucose, 0.1% casamino acids, and 1 mg/l thiamine, and at an initial pH of 4.80.** *E. coli* MG1655 WT and *gadA/B*, and supplementation with glutamate **(A)**, and *E. coli* MG1655 WT and Δ*cadA*, and supplementation with lysine **(B)**. The initial pH of the medium was 4.80. Error bars represent standard deviations of three replicate cultures. The asterisks indicate that the final pH value is significantly different (*p* < 0.05) from the final pH reached with no specific amino acid addition for the same strain.

The addition of arginine or ornithine to the acidified M9 medium did not result in growth improvement of *E. coli* MG1655 at initial pH 4.80 and also did not affect the final pH values of the cultures. On the other hand, the addition of lysine, and to a smaller extent glutamate, enhanced growth and also resulted in a significantly higher final pH value. To further investigate whether the corresponding decarboxylase enzymes are involved in this growth improvement, the experiment was repeated with *E. coli* MG1655 strains deficient in the decarboxylases of the AR systems 2, 3, 4, and 5 (*gadA/B*, *adiA*, Δ*cadA*, and Δ*speF*, respectively) (**Figure 2**). The growth curves and final pH values of the *adiA* and Δ*speF* mutants, with or without addition of arginine or ornithine, respectively, were not significantly different from those of the wild-type strain (data not shown). Further, a small growth improvement and pH increase was observed when glutamate was supplied to cultures of the *gadA/B* mutant, but this was not different from the wild-type strain, indicating that the stimulating effect of glutamate on growth at pH 4.80 was not due to its decarboxylation. On the other hand, supplementation with lysine did no longer improve growth and increase final pH in the Δ*cadA* mutant (**Figure 2**), suggesting that the low pH growth enhancing effect of lysine on *E. coli* is due to lysine decarboxylation by CadA.

**FIGURE 3:**
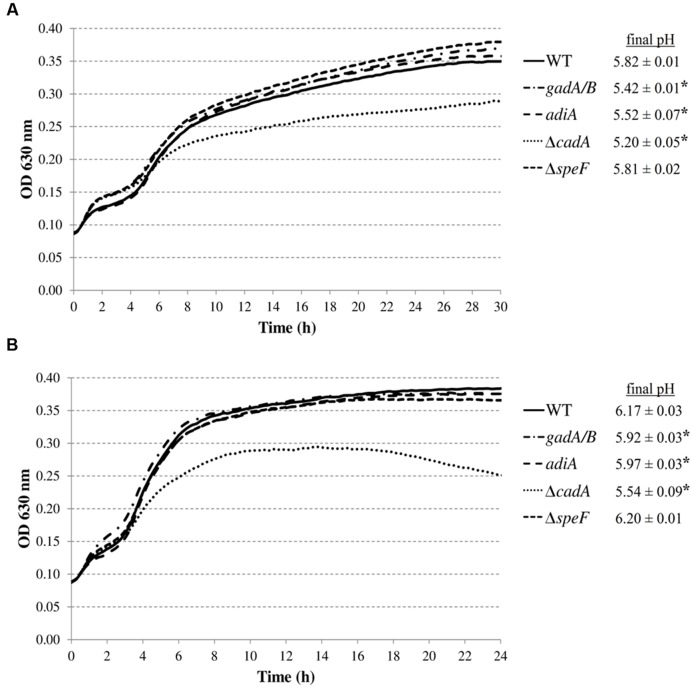
**Growth of *E. coli* MG1655 WT, *gadA/B*, *adiA*, Δ*cadA*, and Δ*speF* in LB acidified with HCl to pH 4.40 (A) or pH 4.60 (B) at 37°C.** Growth curves were averaged across three replicate cultures. The asterisks indicate that the final pH value is significantly different (*p* < 0.05) from that of the wild-type strain.

Subsequently, we investigated whether the decarboxylase enzymes play a role to support growth of *E. coli* MG1655 in a complex medium (LB) at low pH (**Figure 3**). Knockout of *gadA/B*, *adiA*, or *speF* did not result in diminished growth in LB at pH 4.40 or 4.60. However, the final pH values for the *gadA/B* and *adiA* mutants were significantly lower than those of the wild-type strain, indicating that the glutamate and arginine decarboxylases are active during growth of MG1655 under these conditions. Deletion of *cadA* clearly impaired growth in acidified LB and resulted in the lowest final pH values. Finally, deletion of *speF* did not influence *E. coli* growth or final pH.

### Genome-Wide Screening for Mutants of *E. coli* MG1655 Affected in Growth in LB at Low pH

As an open approach to identify novel genes involved in moderately low pH growth, a random transposon insertion mutant library of *E. coli* MG1655 was constructed and screened. To identify suitable screening conditions, the wild-type strain was first grown in LB acidified with HCl to different initial pH values (data not shown). This screening was done at 30°C, because, as mentioned before, this makes the selection more relaxed than 37°C and thus ensures that only the most sensitive mutants are picked up.

The lowest pH at which growth was observed in this experiment was 4.20, and the pH for this culture increased to 5.10 after 72 h. From pH 4.20 to 4.50, the growth rates and final OD_630_ increased rapidly, while a further increase of pH above 4.50 had comparatively less effect on growth. Based on these observations, a pH value of 4.50 was chosen for the screening in combination with an incubation time of 24 h. Although this pH value is only slightly above the pH_min_ under these conditions, the wild-type strain can grow relatively well at this pH value and therefore this combination of pH and time should be suited to select mutants affected in growth at low pH.

Eleven out of the 8544 mutants tested remained below an OD_600_ of 0.100 after 24 h incubation in LB at pH 4.50. Determination of transposon insertion sites revealed six different genes: *mnmE* (1x), *leuO* (5x), *apaH* (1x), *waaC* (1x), *epmA* (1x), and *tatC* (2x). The functions of these genes and the exact position of the transposons are listed in **Table 3**. In all cases except for *leuO*, the transposons were inserted into the open reading frame of the corresponding genes. In all five *leuO* mutants, which were retrieved from independent mutagenesis experiments, the transposon was inserted 26 bp upstream of the *leuO* open reading frame. Although the transposase used in this study has an altered target specificity, exhibiting a much lower degree of insertion specificity than the wild-type transposase (Kleckner et al., 1991), the occurrence of five inserts at exactly the same insertion site may indicate a so-called hotspot at this position. On the other hand, the two insertions in *tatC* were at different positions in the *tatC* gene.

**Table 3:** Overview of genes affected in the transposon insertion mutants of which the growth was impaired in LB pH 4.50.

Gene	Position of transposon	Function
*mnmE*	+820	5-methylaminomethyl-2-thiouridine modification of tRNA
*leuO^∗^*	-26	DNA-binding transcriptional dual regulator
*apaH*	+614	Diadenosine tetraphosphatase
*waaC*	+644	ADP-heptose:LPS heptosyltransferase I
*epmA*	+259	EF-*P*-lysine lysyltransferase
*tatC*	+23; +441	Export of folded proteins across the cytoplasmic membrane

*The position of the transposon gives the nucleatide after which the rtansposon was inserted, starting from the first base of the start codon. In the case of leuO, the insertion was 26 bp upstream of the leuO open reading frame*.

### Construction of Deletion Mutants and Complementation

**FIGURE 4:**
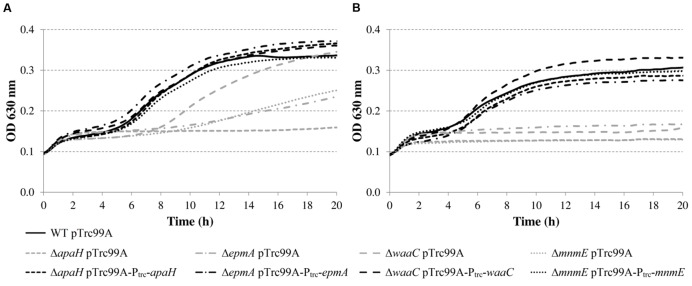
**Growth of *E. coli* MG1655 WT containing pTrc99A, deletion mutants containing pTrc99A, and deletion mutants containing complementation plasmids in LB pH 4.50 at 30°C (A) or 37°C (B).** Growth curves were averaged across three replicate cultures.

To exclude the possibility that these acid-sensitive phenotypes were due to polar genetic effects of the transposon or due to unrelated secondary mutations, *de novo* deletion mutants were made in *E. coli* MG1655. However, the *tatC* mutant was excluded from further analysis, since the *E. coli* K-12 chromosome encodes at least 36 polypeptides that are known or predicted substrates for export by the twin-arginine translocation (Tat) system (TatABCE) (Berks et al., 2005), and inactivation of this system probably has pleiotropic effects on the *E. coli* physiology. The OD_630_ growth curves of these deletion mutants confirmed their reduced growth in LB at pH 4.50, except for the Δ*leuO* mutant, which showed WT growth (data not shown). Since the transposon of the originally isolated mutant was inserted 26 bases upstream of the start codon, we assume it did not completely knock out *leuO* gene function, but may have modified its expression level or regulation. Subsequently, expression constructs derived from the pTrc99A plasmid were made for complementation and introduced into the corresponding deletion mutants (except for *leuO*). The deletion mutants harboring the empty pTrc99A plasmid or the complementation plasmid were then grown in LB at pH 4.50 at 30 or 37°C (**Figure 4**), and the OD_630_ growth curves show that complementation effectively restored moderately low pH growth to wild-type levels.

**FIGURE 5:**
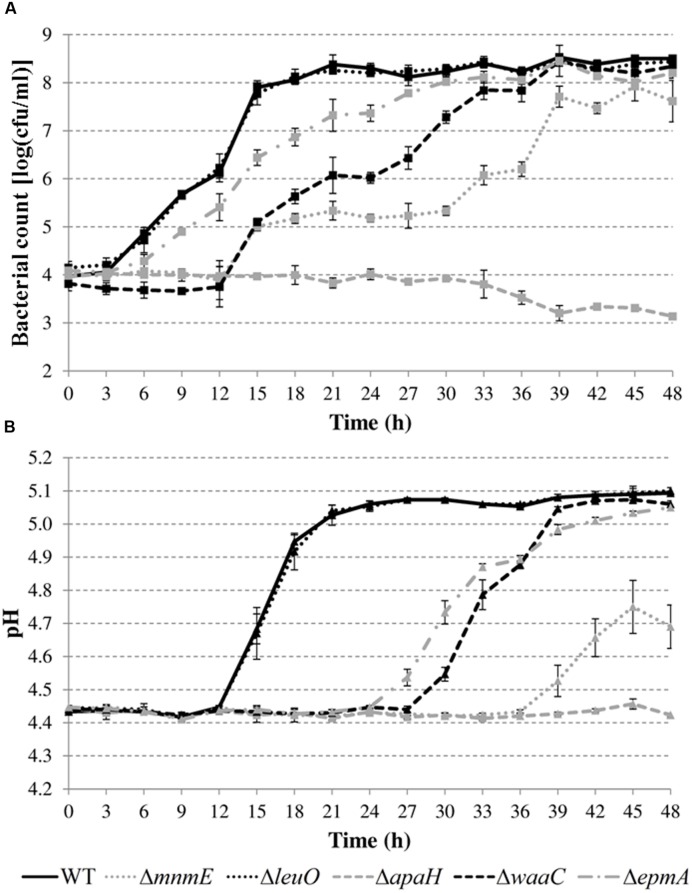
**Cell numbers (A) and pH values (B) during growth of *E. coli* MG1655 WT and deletion mutants in LB pH 4.40 at 30°C.** Error bars represent standard deviations for three independent replicates. The corresponding growth parameters are shown in **Table 4**.

Next, growth of the mutants in acidified LB was evaluated in more detail by determining plate counts and medium pH every 3 h (**Figure 5**). The growth parameter estimates for the initial (*y*_0_) and final (*y*_max_) bacterial cell density, the maximum specific growth rate (μ_max_), and the lag time (*t*_lag_) are shown in **Table 4**.

**Table 4:** Parameter estimates for the initial (*y*_0_) and final (*y*_max_) bacterial cell density, the maximum specific growth rate (μ_max_), and the lag time (*t*_lag_) during growth of *E. coli* MG1655 WT and deletion mutants in acidified LB (initial pH around 4.4) at 30°C for 48 h (growth curves in **Figure 4**).

Growth parameter	WT	Δ*mnmE*	Δ*leuO*	Δ*waaC*	Δ*epmA*
*y*_0_	3.95 ± 0.06	4.02 ± 0.08	4.15 ± 0.16	3.62 ± 0.05^∗^	3.98 ± 0.07
*y*_max_	8.36 ± 0.03	7.93 ± 0.05^∗^	8.32 ± 0.02	8.33 ± 0.09	8.12 ± 0.10^∗^
μ_max_	0.76 ± 0.06	0.26 ± 0.02^∗^	0.80 ± 0.06	0.40 ± 0.02^∗^	0.47 ± 0.05^∗^
*t*_lag_	3.92 ± 0.61	12.76 ± 1.67^∗^	5.00 ± 1.20	8.82 ± 1.03^∗^	4.50 ± 0.73

*Values are means ± standard deviations (n = 3). The asterisks indicate that the parameter value is significantly different (p < 0.05) from that of the wild-type strain.*

The results show that the cell numbers and the pH values for the wild-type and the Δ*leuO* mutant evolved in a similar way, and that the growth parameters for both strains were not significantly different. The Δ*apaH* mutant was not able to grow at this pH (as already evident from **Figure 4**) and its cell numbers decreased slightly, reaching around 3.1 log(cfu/ml) after 48 h. The remaining three mutants had a significantly slower growth rate (μ_max_), and, in addition, the Δ*mnmE* and Δ*epmA* mutants had slightly lower maximal cell densities (*y*_max_), while the Δ*waaC* and Δ*mnmE* mutants exhibited a significantly longer lag phase (*t*_lag_) than the wild-type (**Table 4**). Interestingly, all strains that initiated growth did so without increasing medium pH initially. Only when cell numbers exceeded around 7.0 log(cfu/ml), medium pH started increasing until early stationary phase. The maximum pH value reached by all strains that started growing, except for the Δ*mnmE* mutant, was around 5.10 and this pH value remained unchanged during stationary phase (**Figure 5**).

### Growth of Mutants in Acidic Foods

**FIGURE 6:**
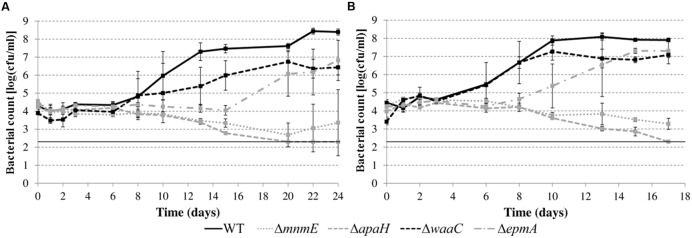
**Growth of *E. coli* MG1655 WT and deletion mutants in apple juice pH 4.60 (A) and tomato juice pH 4.80 (B) at 20°C.** Error bars represent standard deviations of three indemendent replicate cultures. The line at 2.3 log(cfu/ml) represents the lower detection limit.

In a subsequent step, we investigated growth of the deletion mutants (except the Δ*leuO* mutant) in apple juice (pH 4.60) and tomato juice (pH 4.80) (**Figure 6**). While previous experiments in LB were conducted at 30 or 37°C, the experiments in juice were done at 20°C to simulate possible food storage conditions. All the mutants also showed growth defects in these acidic foods but the nature and relative magnitude of the defects was different in these foods than in acidified LB (**Figure 5**) for some mutants. For example, while the Δ*waaC* mutant showed an extended lag phase in acidified LB and the Δ*epmA* mutant did not, this was reversed in the juices. Another example is the Δ*mnmE* mutant, which was able to grow in acidified LB but was slowly inactivated in the juices. The Δ*apaH* mutant showed similar behavior in acidified LB and in the juices, being inactivated in all situations. Also in contrast to growth in acidified LB, bacterial growth in acidic juices did not result in an extracellular pH increase. Instead, the juice pH remained almost constant (maximal variation was 0.04 pH units) for all strains during the entire experiment (data not shown).

### Survival of Mutants in Acid Challenge Assay in Simulated Gastric Fluid

**FIGURE 7:**
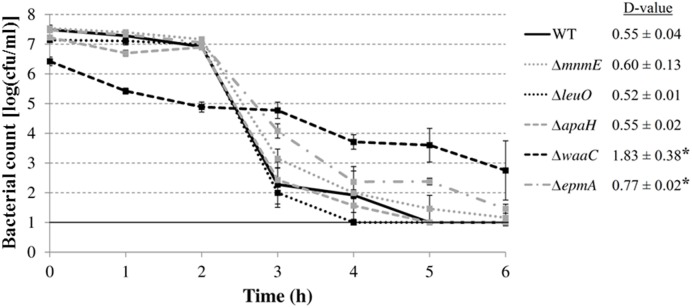
**Survival of *E. coli* MG1655 WT and deletion mutants at 37°C in simulated gastric fluid pH 2.50.** Error bars represent standard deviations of three independent replicate experiments. The line at 1.0 log(cfu/ml) represents the detection limit. *D*-values (in h) are shown next to the figure and are expressed as means ± standard deviations. Asterisks indicate significant differences (*p* < 0.05) with the wild-type strain.

Finally, we investigated whether the mutations rendered *E. coli* MG1655 also more sensitive to inactivation at extremely low pH. Therefore, the AR of the deletion mutants was examined in a simulated gastric fluid (pH 2.50) at 37°C (**Figure 7**). The survival data were fitted and *D*-values were calculated from the linear part. All curves, except that of the Δ*waaC* mutant, displayed a shoulder before a substantial decrease was apparent. Unexpectedly, the Δ*epmA* and Δ*waaC* mutants were more acid resistant than the wild-type strain, showing significantly higher D-values under these conditions, while the other genes did not influence survival of *E. coli* MG1655 in the simulated gastric fluid.

## Discussion

### Amino Acid Decarboxylase Systems

The best described AR mechanisms in *Enterobacteriaceae* rely on proton consumption during amino acid decarboxylation. In *E. coli*, four AR systems (AR 2, 3, 4, and 5) have been described in which a particular amino acid (glutamate, arginine, lysine, or ornithine, respectively) is converted to its corresponding amine (GABA, agmatine, cadaverine, or putrescine, respectively) by a cytoplasmic decarboxylase (the GadA and GadB isozymes, AdiA, CadA, and SpeF, respectively), followed by the exchange of the decarboxylated products for new amino acid substrates via a cognate inner membrane-bound antiporter (GadC, AdiC, CadB, and PotE, respectively) (Zhao and Houry, 2010). The strength of these systems to protect against extreme acid stress has been correlated to the pH optima of the decarboxylase enzymes, being 3.7-3.8, 4.9-5.2, 5.7, and 7.0, respectively (Kanjee and Houry, 2013). Thus, the glutamate-dependent AR system is the most potent AR system in *E. coli*, followed by the arginine- and lysine-dependent AR systems (Iyer et al., 2003; Diez-Gonzalez and Karaibrahimoglu, 2004). The ornithine-dependent AR system has been less well studied and plays only a minor role during survival of an extreme acid challenge. For example, in *E. coli*, only glutamate, arginine, and lysine, but not ornithine, supported robust survival at pH 2.5 in a minimal medium (Iyer et al., 2003). Also survival of *Salmonella* was only modestly improved by ornithine in a minimal medium at pH 2.3 (Viala et al., 2011).

In our study, the ability of these systems to enhance growth under moderate acid stress was investigated. We showed that only lysine considerably improved growth of *E. coli* MG1655 at moderately low pH in a minimal medium, and that this effect relies on lysine decarboxylation since it was not observed in a Δ*cadA* mutant (**Figures 1** and **2**). Similarly, only the lysine decarboxylase CadA enhanced growth of *E. coli* MG1655 at moderately low pH in a complex medium (**Figure 3**). Although also the glutamate decarboxylase isozymes GadA and GadB and the arginine decarboxylase AdiA provoked a slight pH increase during growth in complex medium at moderately low pH, this effect was insufficient to confer a growth advantage. The relative effectiveness of these decarboxylases to support low pH growth may reflect their pH optima. Since *E. coli* cannot maintain an internal pH of more than two units higher than the external pH (Foster, 2004), the internal pH during growth at an external pH of 4.5 is expected to be around 6.5, which is close to the pH optimum of 5.7 of CadA. However, other factors are also involved, since the ornithine decarboxylase SpeF (pH optimum 7.0) did not contribute to growth under these conditions. This is in contrast to *Salmonella*, in which both lysine and ornithine decarboxylases significantly improved growth at moderately acidic pH under anoxic conditions in a minimal medium supplemented with these amino acids (Viala et al., 2011).

A mutant that can be indirectly linked to the lysine decarboxylase system is the mutant which had the transposon inserted just 26 bp upstream of the *leuO* open reading frame, leaving the putative ribosomal binding site fully intact. Remarkably, insertion of a Tn*10* transposon at exactly the same site has been reported previously, and was shown to result in overexpression of LeuO (Klauck et al., 1997). This might also be the case in our study since deletion of *leuO* did not affect the growth of *E. coli* MG1655 at moderately low pH. Moreover, it has been shown that LeuO overexpression drastically reduces production of CadC, the essential activator for *cadA* induction (Shi and Bennett, 1995), and this might explain its role in growth at low pH. Furthermore, LeuO has also indirectly been linked to AR since it represses the small non-coding RNA *dsrA*, which plays a regulatory role in AR in *E. coli* (Lease et al., 2004). Finally, also the *epmA* gene, discussed in the next section, can be linked to CadA activity.

In conclusion, our results show that the decarboxylase enzymes not only produce better survival at extremely low pH by counteracting intracellular acidification (Richard and Foster, 2004), they also trigger deacidification of the extracellular medium, thereby potentially enhancing growth at moderately low pH.

### Aminoacyl-tRNA Synthetases and Translation Elongation

The mutant with the strongest growth defect at low pH was an *apaH* mutant. This mutant was not able to grow in LB at pH 4.50, while its growth rate was only slightly less than that of the wild-type at neutral pH (data not shown). The *apaH* gene encodes diadenosine tetraphosphatase, which hydrolyzes 5’,5”’-P^1^, P^4^-diadenosine tetraphosphate (AppppA) to two molecules of adenosine diphosphate (ADP) (Guranowski et al., 1983). AppppA is rapidly synthesized by aminoacyl-tRNA synthetases when *E. coli* is exposed to heat shock or oxidative stress and may serve as a modulator of these stress responses (Johnstone and Farr, 1991). It has been shown that *apaH* mutants, which have high basal levels of AppppA, are sensitive to killing by heat and oxidative stress, and are unable to grow at 43°C (Johnstone and Farr, 1991). AppppA binds to several proteins in *E. coli*, including DnaK, GroEL, and ClpB, and AppppA binding may inhibit DnaK and/or GroEL functions that are required for survival of thermal stress. Thus, the regulation of the AppppA pool is important for the bacterial response to conditions of stress and AppppA may assist the return of cells to normal growth conditions following stress, but constitutively high levels of AppppA may inhibit the function of stress proteins such as DnaK before they have fulfilled their role under that stress (Johnstone and Farr, 1991; McLennan et al., 2001). Our results suggest that AppppA is also involved in cellular adaptation to acid stress. Since the cytoplasmic chaperones DnaK and GroEL have also been linked to counteracting acid stress in several bacteria, such as *S. enterica* and *H. pylori*, a similar mechanism may also account for the role of AppppA under acid stress (Bearson et al., 2006).

Another protein that was found in our work to play a role during growth at low pH was EpmA. EpmA specifically aminoacylates the translation elongation factor P (EF-P) at a conserved lysine residue with β-lysine. This posttranslational modification activates EF-P to function in translation elongation of a particular subset of mRNAs (Bullwinkle et al., 2013). It has been demonstrated that EF-P enhances the translation of polyproline-containing proteins by alleviating ribosome stalling at polyproline stretches (Ude et al., 2013). One such protein is CadC, a membrane-integrated transcriptional regulator that both senses external pH and activates expression of the *cadBA* operon at low external pH. EF-P was shown to be required for translation of CadC and a deletion mutant of *epmA* lacked CadA activity (Ude et al., 2013), which may account for the diminished growth at moderately low pH in our experiments. Interestingly, EpmA is an aminoacyl-tRNA synthetase paralog, showing sequence similarity to the lysyl-tRNA synthetases LysS and LysU. Whereas LysS is constitutively expressed, LysU is overexpressed under extreme physiological conditions, such as heat shock, and is the most effective AppppA synthetase, producing 80% of total AppppA in *E. coli* cell extracts (Charlier and Sanchez, 1987). In addition to high temperature, LysU has also been shown to be upregulated at moderately low pH (Hayes et al., 2006). Interestingly, the activity of LysU has been shown to be less sensitive than that of LysS to the competitive inhibitor cadaverine, the decarboxylation product of lysine, suggesting that LysU plays a more important role under physiological conditions causing cadaverine accumulation, such as acid stress (Brevet et al., 1995). Although EpmA is homologous to the catalytic core of the lysyl-tRNA synthetases, it lacks the tRNA aminoacylation activity due to absence of the tRNA anticodon-binding domain. Nevertheless, EpmA retains the ability to activate L-lysine by formation of the lysyl-adenylate intermediate (Ambrogelly et al., 2010), which is also the first step in AppppA synthesis. However, it is not known whether EpmA can also contribute to AppppA production.

The screening also yielded a gene that is involved in tRNA modification, *mnmE*. Together with MnmG, MnmE forms a complex that adds an aminomethyl or carboxymethylaminomethyl group to position 5 of the anticodon wobble uridine (U_34_) using methylene-tetrahydrofolate and ammonium or glycine as donors, respectively (Moukadiri et al., 2014). Approximately 85 different modifications of tRNA molecules have been documented, and they are thought to be important for maintaining tRNA structure, and for specific recognition of tRNA molecules by their cognate aminoacyl-tRNA synthetases. Besides, the idea that tRNA modifications can take on second-order regulatory functions, especially in response to stress conditions, has recently emerged. Some recent studies have linked tRNA modification to the control of gene expression at the level of translation in response to environmental stresses. For example, Begley et al. (2007) have shown that the tRNA methyltransferase 9 (Trm9) of *Saccharomyces cerevisiae*, which catalyzes the last step (methylation) in the methylcarbonylmethyl (mcm) modification on position 5 of the uridine wobble base (U_34_) of tRNA^Arg^_mcm5UCU_ and tRNA^Glu^_mcm5s2UUC_, prevents cell death during methyl methanesulfonate exposure via translational enhancement of DNA damage response proteins whose genes are overrepresented with the AGA (Arg) and GAA (Glu) codons. This indicates that tRNA modifications indirectly help coordinate DNA repair. Also in *S. cerevisiae*, it has been demonstrated that the modifications 2’-*O*-methylcytosine (Cm), 5-methylcytosine (m^5^C), and *N*^2^,*N*^2^-dimethylguanosine (m^2^_2_G) increase following exposure to hydrogen peroxide and that loss of the methyltransferase enzymes catalyzing the formation of these modified nucleosides causes hypersensitivity to hydrogen peroxide (Chan et al., 2010). Thus, tRNA modifications can dynamically change in response to stress and may be critical features of the cellular stress response. Furthermore, exposing *S. cerevisiae* to hydrogen peroxide resulted in a Trm4 methyltransferase-dependent increase in the incorporation of m^5^C in tRNA^Leu^_m5CAA_, which causes selective translation of mRNA from genes that are enriched in the TTG codon for leucine (Chan et al., 2012). Cells may thus respond and adapt to environmental stresses by reprogramming of tRNA modifications, thereby promoting the selective translation of codon-biased mRNAs for critical stress response proteins (Dedon and Begley, 2014). Our work extends these observations by linking t-RNA modification by MnmE to growth at low pH in *E. coli*.

### Cell Envelope

The *waaC* gene which was picked up in our screening is involved in the synthesis of lipolpolysaccharide (LPS). WaaC adds ADP-L-*glycero*-D-*manno*-heptose on the inner 3-deoxy-D-*manno*-octulosonic acid (KDO) residue of the LPS inner core with the release of ADP (Kadrmas and Raetz, 1998). Knockout of *waaC* results in a heptoseless truncation of the LPS and a so-called deep-rough phenotype, characterized by mucoid colonies phenotype due to production of a colanic acid capsular polysaccharide (Joloba et al., 2004). This phenotype was clearly visible when these mutants were grown on agar plates in our experiments. The production of this polysaccharide might also be the reason for their higher final OD values compared to the wild-type in some growth experiments (**Figure 4B**). Bielecki et al. (1982) used chemical mutagenesis to isolate mutants of *E. coli* K-12 that were not able to grow on LB agar plates acidified with HCl to pH 5.4. Four mutants were picked up and a number of altered phenotypes (phage and detergent sensitivities, leakage of periplasmic proteins) suggested that these mutants probably belonged to a group of deep-rough mutants defective in their LPS. Thus, the capacity of the outer membrane to form a proton barrier is probably reduced by lesions in the LPS structure (Rowbury, 2004). Furthermore, Barua et al. (2002) isolated five transposon insertion mutants of *E. coli* O157:H7 that showed poor or no growth on LB-MES agar with 12 mM acetic acid (pH 5.4). Two of them had an inactivated *waaG* gene, also causing a deep-rough phenotype. The other three genes (*fcl*, *wecA*, and *wecB*) were involved in the biosynthesis of the surface *O*-polysaccharide and/or enterobacterial common antigen.

Interestingly, a *waaC* mutant has been shown to be unable to express the outer membrane protein A (OmpA) (Beher and Schnaitman, 1981). OmpA is one of the most abundant proteins in the outer membrane of *E. coli* and is believed to be a nonspecific diffusion channel, allowing the passage of various small solutes, but its physiological function is unclear. OmpA is induced by acid in *E. coli* and has previously been linked with AR in *E. coli* since an *ompA* mutant was more readily killed by lethal acid stress (pH 3.8; 60 mM acetic acid) than its parent strain (Wang, 2002). OmpA is also important for the structural integrity of the cell envelope, and loss of OmpA probably leads to increased penetration of protons or undissociated acids through the outer membrane due to reduced barrier properties (Rowbury, 2004).

### Efficiency of Mutant Screening

The discussion above suggests that besides the six mutants isolated in this work, several additional mutants are predicted to have a low pH growth phenotype, like *cadA*, *cadC*, *ompA*, *mnmG*, *fcl*, *wecA*, *wecB*. Assuming random transposition of the mini-Tn10 transposon, the probability *P* that the library contains at least one insertion in each (non-essential) gene can be calculated from *N* = ln(1-*P*)/ln(1-a/b), with *N* = number of mutants in the library, *a* = average size of a gene (1000 bp), and *b* = genome size (excluding all essential genes). For our library of 8544 mutants, this gives a probability of 86% that any particular non-essential gene is represented. It seems therefore that the screen picked up less mutants than would be expected. This may be partly explained by non-randomness of the tranposon and the fact that inserts in the 3′ end of a gene do not necessarily knock out protein function. However, probably a more important explanation is that threshold set for isolation of mutants (OD_600_ < 0.100 after 24 h incubation in LB at pH 4.50) filtered out only the most sensitive mutants. A screen based on recording full growth curves (as opposed to end point measurement) would most likely reveal additional, more subtle mutations.

## Conclusion

By identifying genes required for growth at moderately low pH, this work has yielded new insights in the cellular mechanisms used in *E. coli* to cope with mild acid stress. The lysine decarboxylase system, but not other amino acid decarboxylases known to contribute to survival of extreme acid stress, was demonstrated to support growth at moderately low pH. In line with previous findings, the integrity of the outer membrane was also shown to be important for growth at low pH. In addition, tRNA modification and diadenosine tetraphosphate hydrolysis were identified for the first time to be required for low pH growth. Except for the lysine decarboxylase, the cellular functions supporting low pH growth did not support survival of extreme low pH. This work will contribute to a better understanding of microbial survival and growth in mildly acidic foods.

## Author Contributions

CM: Conception of research project, research strategy, interpretation of results, final stages of writing of manuscript. BV: Research strategy, experimental work, interpretation of results, writing of manuscript. AA: Conception of research project, research strategy, interpretation of results

## Conflict of Interest Statement

The authors declare that the research was conducted in the absence of any commercial or financial relationships that could be construed as a potential conflict of interest.
